# Man With Recurrent Epigastric Pain

**DOI:** 10.1016/j.acepjo.2025.100054

**Published:** 2025-02-04

**Authors:** Chew Kiat Yeoh, Nicole Mun Teng Cheung, Isabel En Yun Lum

**Affiliations:** 1 Department of Emergency Medicine, National University Hospital, National University Health System, Singapore; 2 Department of Emergency Medicine, Ng Teng Fong General Hospital, National University Health System, Singapore

**Keywords:** choledochal cyst, abdominal pain, POCUS

## 1 Patient Presentation

**Figure 1:**
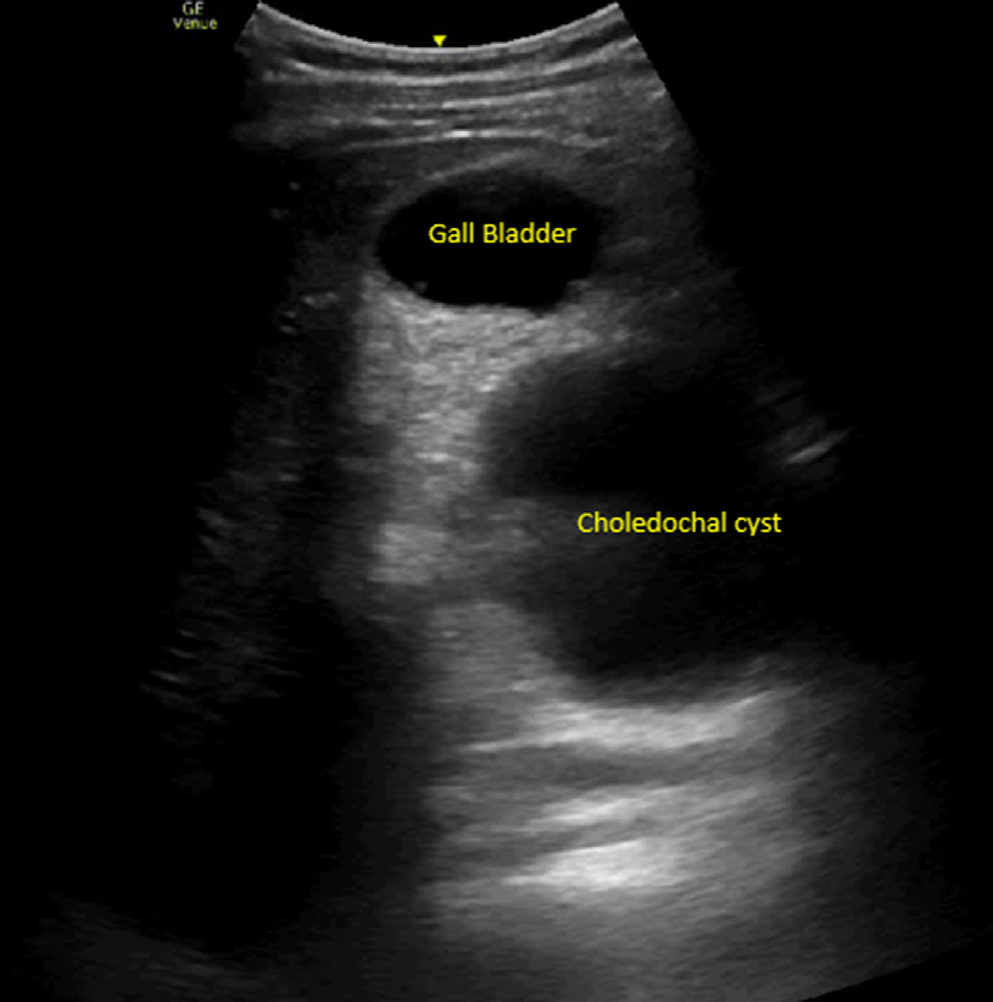
Point-of-care ultrasound demonstrated a normal gall bladder and large choledochal cyst. The choledochal cyst is easily mistaken as part of the gall bladder in this view.

**Figure 2:**
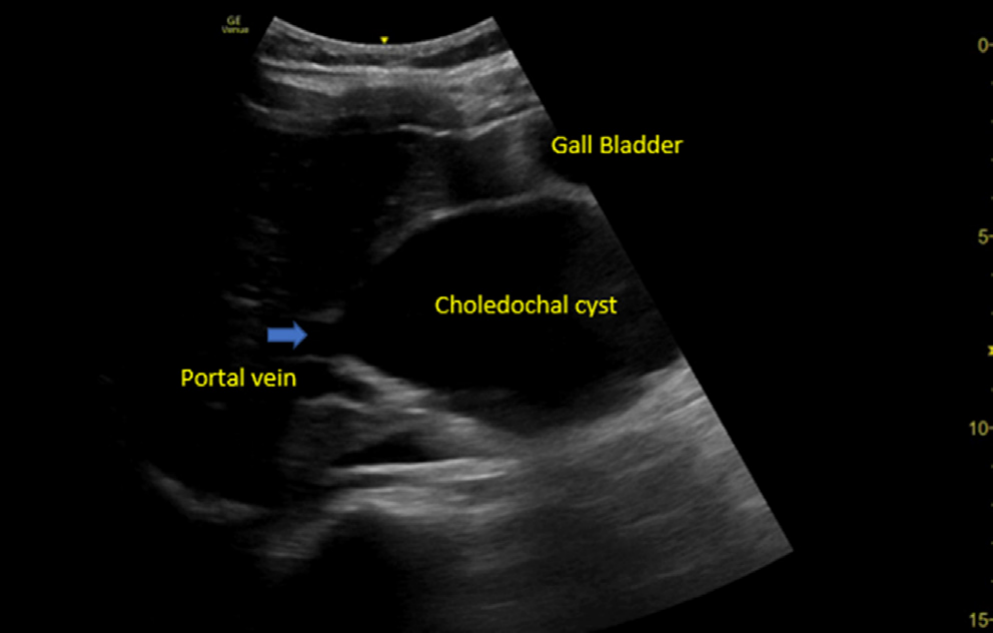
Point-of-care ultrasound showed an evaluation of the common bile duct (blue arrow), which lies above the portal vein and continues with the choledochal cyst.

**Figure 3:**
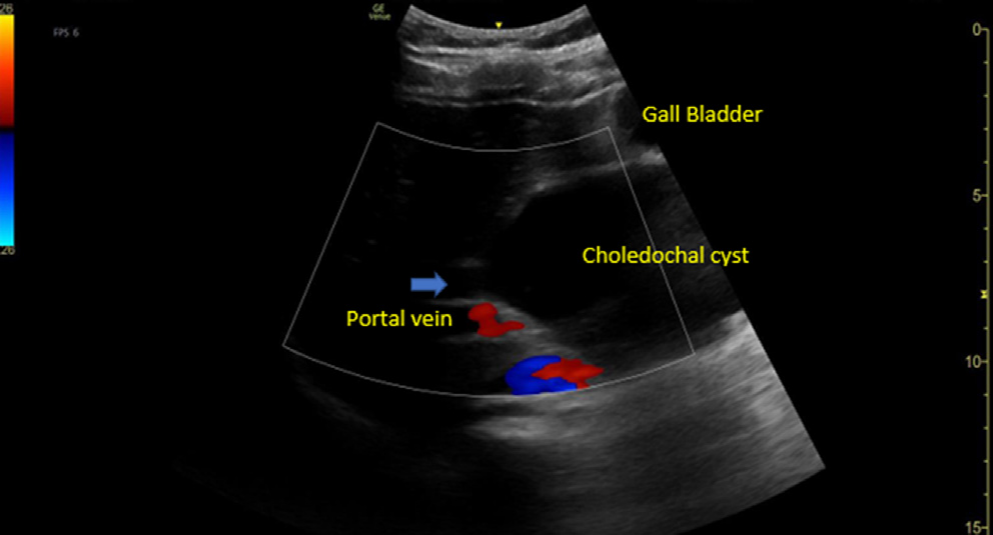
Point-of-care ultrasound showed an absence of color flow in the common bile duct (blue arrow) and choledochal cyst.

A 28-year-old man presented to the emergency department with recurrent epigastric pain for the past year. He was treated for biliary colic 2 days ago as a point-of-care ultrasound (POCUS) showed stones in the gall bladder. His vital signs were normal. Physical examination revealed tenderness over the right hypochondrium and epigastric region. Laboratory investigation revealed an obstructive cholestasis picture with transaminitis. POCUS was repeated (Figure 1, Figure 2, Figure 3).

## 2 Diagnosis: Choledochal Cyst

**Figure 4:**
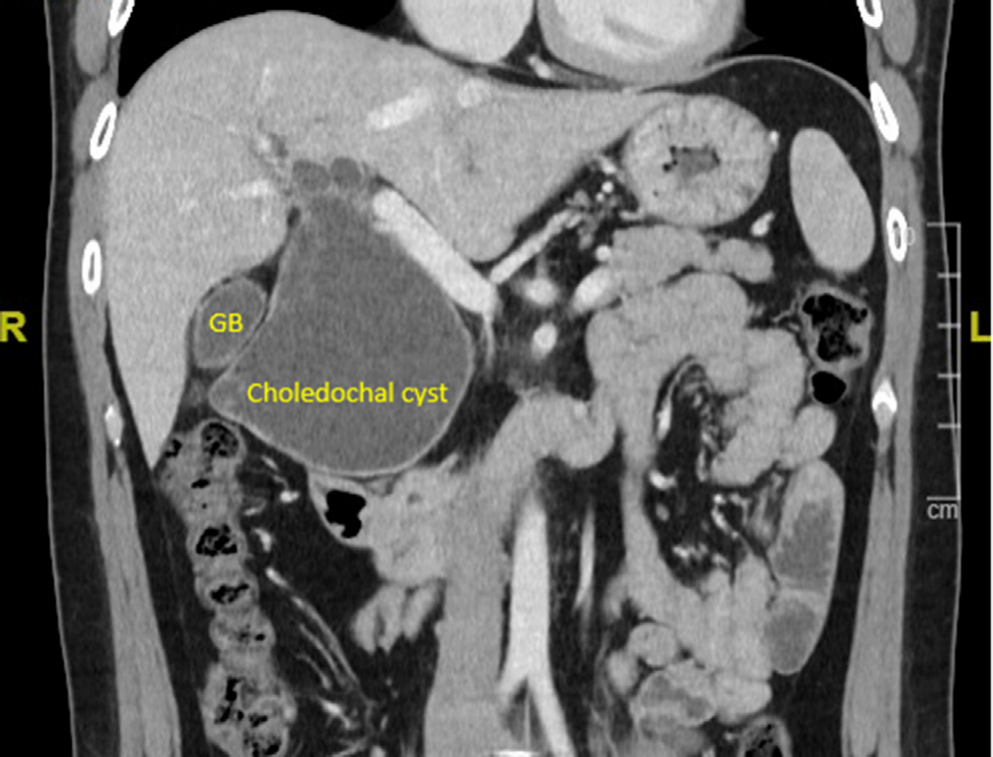
Computed tomography of the abdomen and pelvis showed a large choledochal cyst and normal GB. GB, gall bladder.

Computed tomography of the abdomen and pelvis was performed and confirmed a large 9.0 × 8.0 × 7.0 cm choledochal cyst with extension to bilateral intrahepatic ducts (Fig 4). He was admitted and underwent choledochal cyst excision, cholecystectomy, and hepaticojejunostomy. He was discharged well after 2 weeks of hospitalization.

Choledochal cysts are rare cystic dilation of the intrahepatic and/or extrahepatic bile ducts. The incidence is rare and usually diagnosed in childhood. They mostly present with right upper quadrant abdominal mass, jaundice, and abdominal pain.^1^ POCUS is often used as a part of the quick diagnostic evaluation for patients with suspected hepatobiliary pathology.^2^ A choledochal cyst is commonly mistaken as a gall bladder or liver cyst if the common bile duct is not included as part of the ultrasound evaluation.

## Funding and Support

No grants or other financial support were received.

## Conflict of Interest

All authors have affirmed they have no conflicts of interest to declare.
